# The course of dry eye after phacoemulsification surgery

**DOI:** 10.1186/s12886-015-0058-3

**Published:** 2015-06-30

**Authors:** Servet Cetinkaya, Emine Mestan, Nursen Oncel Acir, Yasemin Fatma Cetinkaya, Zeynep Dadaci, Halil Ibrahim Yener

**Affiliations:** Ophthalmology Clinics, Turkish Red Crescent Hospital, Konya, Turkey; Department of Neurology, Dumlupinar University, Faculty of Medicine, Kutahya, Turkey; Department of Ophthalmology, Faculty of Medicine, Mevlana University, Konya, Turkey; Department of Ophthalmology, Ataturk Training and Research Hospital, Ankara, Turkey; Konya Eye Centre Hospital, Konya, Turkey; Turkish Red Crescent Hospital (Kizilay Hastanesi), Ophthalmology Clinics, Sukran Mah. Taskapu Medrese Sok. No:15, Meram, 42200 Konya, Turkey

**Keywords:** Phacoemulsification, Dry eye, Break-up Time, Schirmer

## Abstract

**Background:**

The aim of this retrospective study was to evaluate the course of dry eye syndrome after phacoemulsification surgery.

**Methods:**

One hundred and ninety-two eyes of 96 patients (30 males, 66 females) with chronic dry eye syndrome and cataract, who had undergone phacoemulsification surgery were enrolled in this study.

**Results:**

Their mean age was 68.46 ± 8.14 standard deviation (SD) (range 56–83) years . Thirty of them (31 %) were males and 66 (69 %) were females. Ocular Surface Disease Index (OSDI) questionnaire scores increased postoperatively, but arrived preoperative levels at the end of 3rd month following the surgery. Fluorescein staining patterns according to Oxford Schema got worse postoperatively, however after postoperative 3rd month they got better and resembled preoperative patterns. The mean postoperative 1st day, 1st week and 1st month Break-up Time (BUT) values were significantly lower than preoperative BUT value (*P* < 0.001, *P* < 0.001, *P* < 0.001), however 3rd month, 6th month, 1st year and 2nd year values were not significantly different from preoperative value (*P* = 0.441, *P* = 0.078, *P* = 0.145, *P* = 0.125). The mean postoperative 1st day, 1st week and 1st month Schirmer Test 1 (ST1) values were significantly lower than preoperative ST1 value (*P* < 0.001, *P* < 0.001, *P* < 0.001), however 3rd month, 6th month, 1st year and 2nd year values were not significantly different from preoperative value (*P* = 0.748, *P* = 0.439, *P* = 0.091, *P* = 0.214).

**Conclusion:**

Phacoemulsification surgery may aggravate the signs and symptoms of dry eye and affect dry eye test values in chronic dry eye patients in short-term. However, in long-term, signs and symptoms of dry eye decrease and dry eye test values return to preoperative values.

**Electronic supplementary material:**

The online version of this article (doi:10.1186/s12886-015-0058-3) contains supplementary material, which is available to authorized users.

## Background

Dry eye syndrome is a multifactorial disease characterized by dryness of the ocular surface due to tear deficiency and overevaporation [1, 2]. There are many causes and factors leading to dry eye, including aging, female gender, connective tissue diseases, Diabetes Mellitus, systemic hypertension, contact lens usage, drugs like antihistamines, anticholinergics, antidepressants, oral contraceptives and topical eye drops containing preservatives and ocular diseases like blepharitis, chronic conjunctivitis, meibomitis and pterygium [3–5]. The symptoms observed in dry eye syndrome include dryness, irritation, burning, foreign body sensation, heaviness of the eyelids, redness, reflex lacrimation, ocular pain and fatigue. It may cause punctate keratitis, persistent epithelial defect, filamentary keratopathy, superior limbic keratoconjunctivitis and reduced visual acuity [6, 7].

Some surgical interventions related to anterior segment may also cause dry eye and aggravate the symptoms in pre-existing dry eye, like PRK, LASIK and cataract surgery [8–10].

In this study, we evaluated the course of dry eye syndrome after phacoemulsification surgery.

## Methods

The study protocol was approved by the local ethics commitee (Selcuk University,Faculty of Medicine Ethics Commitee, Konya, Turkey). An informed written consent was obtained from the patients for the cataract surgery. The study was carried out according to the tenets of the Declaration of Helsinki.

One hundred and ninety-two eyes of 96 patients with chronic dry eye syndrome and cataract were enrolled in this study. They had undergone uneventful phacoemulsification and IOL implantation operation between January 2010 and March 2011. Their medical records were evaluated retrospectively. Their mean age was 68.46 ± 8.14 (SD) (56–83) years. Thirty of them (31 %) were male and 66 (69 %) were female. They all had bilateral cataracts. All of the surgeries were performed by a single surgeon (SC). Under subtenon anesthesia, a 2.75 mm clear corneal incision was made. Anterior chamber was filled with a dispersive (hydroxypropylmethylcellulose, Easy Visc, Germany) viscoelastic substance. After continuous curvilinear capsulorhexis, hydrodissection and hydrodelineation was performed, then a sideport entrance was made. The nucleus was removed by using the “divide and conquer” technique (Sovereing Compact, Phacoemulsification System, AMO, USA). The cortex was aspirated with coaxial irrigation/aspiration. The capsular bag was filled with a cohesive (Na Hyaluronate 1.6, Easyluron, Germany) viscoelastic substance. A foldable monofocal posterior chamber IOL (Acriva, VSY, Turkey) was implanted in the capsular bag through an injector system. The viscoelastic material was aspirated completely. The entrances were closed with stromal hydration. After the operation patients used topical antibiotic (Moxifloxacin 0.5 %, Vigamox, Alcon, USA) four times a day for a week and topical steroid (Dexamethasone Na Phosphate 0.1 %, Dexa-sine SE, Liba, USA) six times a day for a week and tapered for subsequent 3 weeks. These two eyedrops did not contain any preservatives. They were all taking artificial tears therapy routinely and 12 of them (12 %) were taking additional topical cyclosporin A. Their therapies were not interrupted due to the surgery. Their full ophthalmological examinations were performed 1 week before the surgery and 1st day, 1st week, 1st month, 3rd month, 6th month, 1st year and 2nd year after the surgery and additionally fluorescein staining, BUT and ST1 without anesthesia were performed owing to the chronic dry eye. OSDI questionnaire was applied 1 week before the surgery and 1st week, 1st month, 3rd month and 6th month after the surgery.

OSDI score was calculated by this formula: Total points of all answered questions x 100/Total number of answered questions x 4. The range of OSDI scores is between 0 and 100. Scores over 25 shows dry eye syndrome. Fluorescein staining was classified according to Oxford Schema (Grade 0 to 5). Grade 2 and greater than this level indicate dry eye syndrome. For BUT, a fluorescein strip was placed in the inferior fornix and the patient was asked to blink several times and with slit lamp biomicroscopy by using cobalt blue filter, the interval between last blink and first appearance of a dry spot or tear film break-up was recorded, and this was repeated three times and the average was determined. Values shorter than 10 s indicate dry eye syndrome. For ST1, Schirmer strip was inserted into the inferior fornix beneath the temporal lid margin, after 5 min, strip was removed and the wetness was measured. Values lower than 5 mm are diagnostic for dry eye syndrome.

### Statistical analysis

For statistical analysis, SPSS version 22 programme was used. For comparison of the data, Chi-square test and Paired *t* test were used. A *P* < 0.05 value was accepted as statistically significant.

## Results

The time of dry eye diagnosis of these patients was approximately 1 to 5 years prior to the surgery. During the time of diagnosis at the first examination, all of the patients had complaints such as burning, stinging, redness, dryness, foreign body sensation, pain and fatigue in their eyes. OSDI scores were between 25 and 50 in 66 (69 %) patients and between 50 and 75 in 30 (31 %) patients. Fluorescein staining, BUT and ST1 tests were performed. Twenty-four eyes (12 %) had grade 4, 42 eyes (22 %) had grade 3, 66 eyes (34 %) had grade 2 and 60 eyes (31 %) had grade 1 staining pattern according to Oxford Schema. BUT values were under 10 s in 138 eyes (72 %) and under 5 s in 54 eyes (28 %). ST1 values were under 5 mm in 150 eyes (78 %) and under 3 mm in 42 eyes (22 %). We commenced topical artificial tears therapy for all of them and additional topical cyclosporin A for 30 of them. Cyclosporin A therapy was ceased and restarted according to the clinical courses of the patients.

**Fig. 1:**
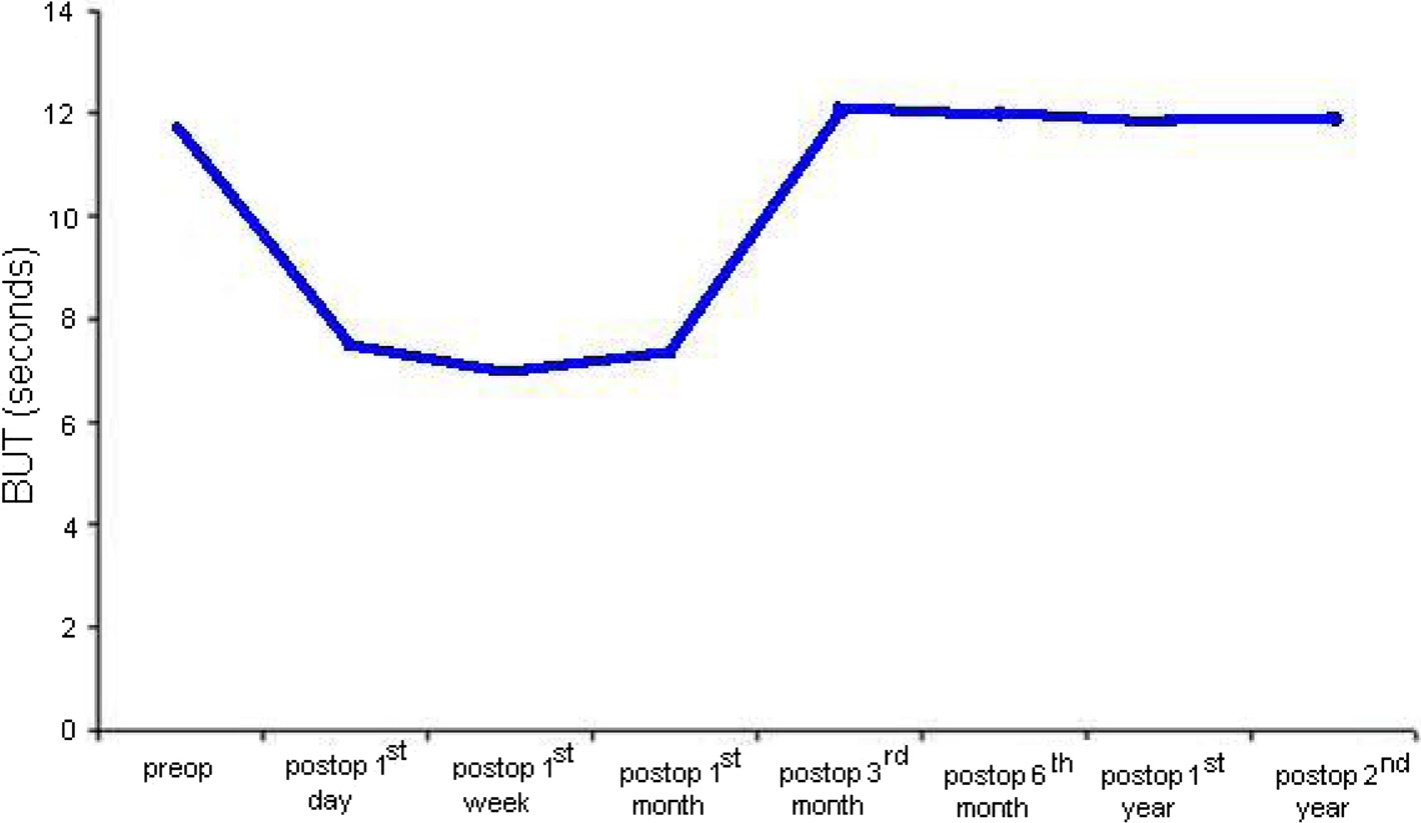
The course of BUT values

The frequency of women were significantly higher than that of men (*P* = 0.003). OSDI scores were under 25 in 87 (91 %) patients and between 25 and 30 in 9 (9 %) patients preoperatively. However, postoperatively in 1st week, it was under 25 in 15 (16 %) patients, between 25 and 30 in 33 (34 %) patients, between 30 and 40 in 21 (22 %) patients and between 40 and 50 in 27 (28 %) patients. In postoperative 1st month, it was under 25 in 30 (31 %) patients, between 25 and 30 in 39 (41 %) patients and between 30 and 40 in 27 (28 %) patients. In postoperative 3rd month it was under 25 in 84 (88 %) patients and between 25 and 30 in 12 (12 %) patients. In postoperative 6th month it was under 25 in 90 (94 %) patients and between 25 and 30 in 6 (6 %) patients. According to Oxford Schema, preoperatively only 15 eyes had grade 2 fluorescein staining (7 %). But postoperatively on 1st day, 36 eyes had grade 2 (18 %), 24 eyes had grade 3 (12 %) and 12 eyes had grade 4 (6 %) fluorescein staining pattern. In 1st week 24 eyes had grade 2 (12 %) and 12 eyes had grade 3 (6 %) staining. In 1st month 18 eyes had grade 2 (9 %) and 6 eyes had grade 3 (3 %) staining. In 3rd month, only 6 eyes had grade 2 staining pattern (3 %). The mean preoperative BUT value was 11.65 ± 2.31 (SD) (7–16) seconds. Postoperative 1st day value was 7.60 ± 1.24 (SD) (5–11), 1st week value 7.03 ± 0.97 (SD) (5–9), 1st month value 7.42 ± 0.79 (SD) (6–8), 3rd month value 11.76 ± 2.08 (SD) (9–16), 6th month value 12.01 ± 2.05 (SD) (9–16), 1st year value 11.85 ± 2,01 (SD) (8–17) and 2nd year value was 11.95 ± 1.92 (SD) (9–17) seconds. In comparison with preoperative value, the 1st day, 1st week and 1st month values were significantly lower (*P* < 0.001, *P* < 0.001,*P* < 0.001), however 3rd month, 6th month, 1st year and 2nd year values were not significantly different from preoperative value (*P* = 0.441, *P* = 0.078, *P* = 0.145, *P* = 0.125). This is shown in Fig. 1.

**Fig. 2:**
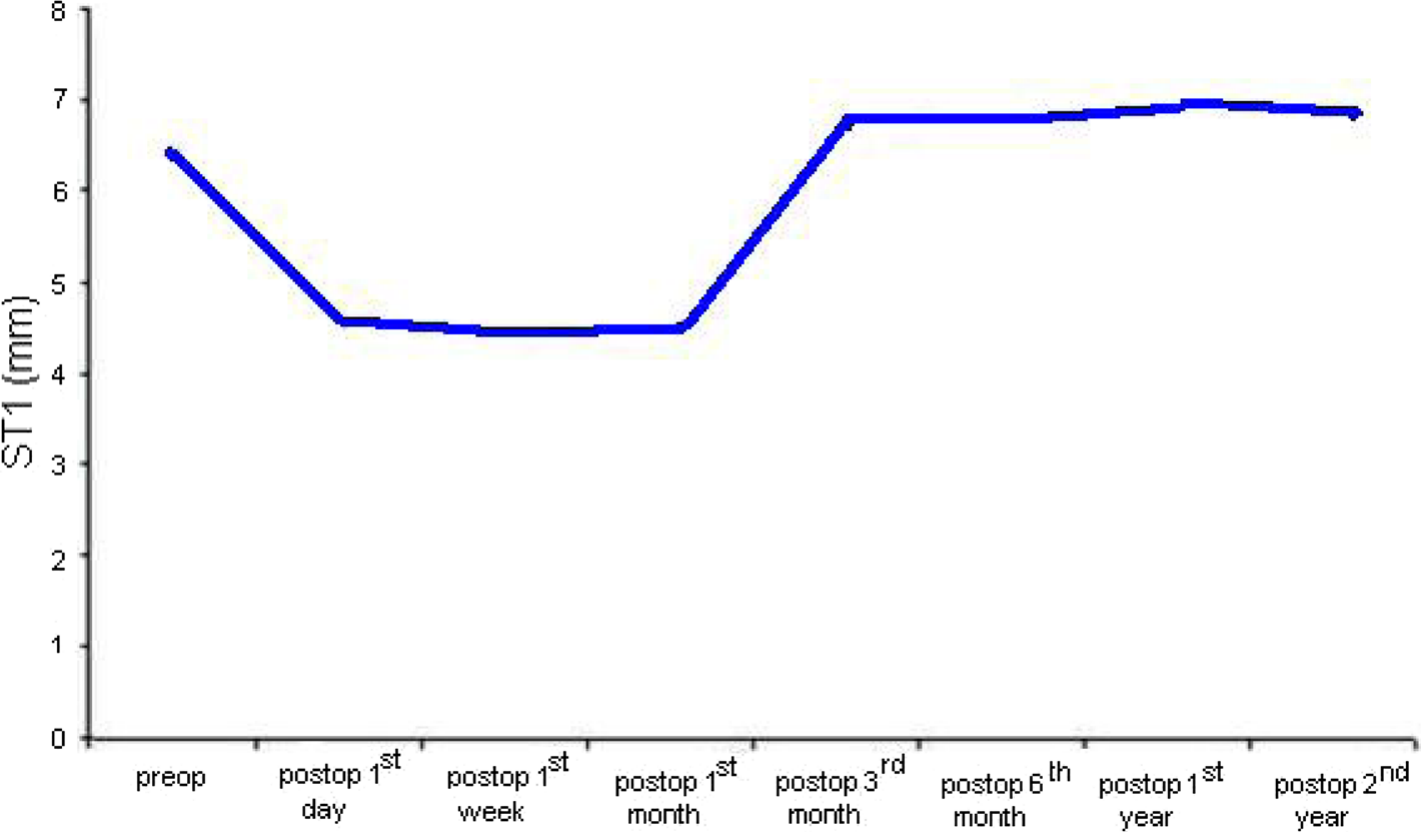
The course of ST1 values

The mean preoperative ST1 value was 6.39 ± 1.42 (SD) (4–9) mm. Postoperative 1st day value was 4.59 ± 1.06 (SD) (3–7), 1st week value 4.45 ± 0.95 (SD) (2–6), 1st month value 4.50 ± 1.00 (SD) (3–6), 3rd month value 6.42 ± 1.31 (SD) (4–9), 6th month value 6.46 ± 1.28 (SD) (4–10), 1st year value 6.59 ± 1.38 (SD) (4–9) and 2nd year value was 6.54 ± 1.29 (SD) (4–9) mm. In comparison with preoperative value, 1st day, 1st week and 1st month values were significantly lower (*P* < 0.001, *P* < 0.001, *P* < 0.001), however 3rd month, 6th month, 1st year and 2nd year values were not significantly different from preoperative value (*P* = 0.748, *P* = 0.439, *P* = 0.091, *P* = 0.214). This is shown in Fig. 2.

By the way, the subjective symptoms of the patients related to dry eye increased postoperatively. But after postoperative 1st month, their complaints decreased gradually. As it was seen; fluorescein staining, BUT and ST1 were impaired up to 1st month postoperatively, however, after 1st month, they improved and they returned to preoperative levels in 3rd month (Additional file 1).

## Discussion

Cornea is innervated by long ciliary nerves of ophthalmic branch of the fifth (trigeminal) nerve. In normal conditions, these nerves send afferent stimuli to brain stem and parasymphathetic and symphathetic signals stimulate lacrimal gland for tear production and secretion [11–13]. For normal blinking and tearing reflexes, intact corneal innervation is necessary. Damage of this circuit causes dry eye. Surgical procedures like PRK, LASIK, extracapsular cataract extraction and phacoemulsification causing denervation of cornea result in decreased blinking and reduction in tear production thus leading to increased epithelial permeability, decreased epithelial metabolic activity and impaired epithelial wound healing [14, 15]. Inflammatory mediators released after corneal incisions may also change the actions of the corneal nerves and reduce corneal sensitivity and result in tear film instability [16, 17]. In healing process, neural growth factor is released to regenerate the subepithelial corneal axon, this process is completed approximately within 1 month and this recovery of the nerves may explain why dry eye signs and symptoms are prominent early after surgery and improve thereafter [16]. This is in accordance with our study. Incision site is larger in LASIK and extracapsular cataract extraction in comparison with phacoemulsification, hence, dry eye signs and symptoms are more prominent and last longer in these patients [18, 19].

Vigorous irrigation of the cornea intraoperatively and ocular surface manipulation deteriorate tear film stability and may reduce goblet cell density and thus cause shortened BUT postoperatively [6, 20]. Exposure to microscope light may also aggravate dry eye symptoms postoperatively [15]. In our study also, BUT values decreased postoperatively.

The use of topical anesthesia, topical eye drops containing preservatives like benzalkonium chloride administered preoperatively and postoperatively may cause tear film instability and decrease the number of mucin expressing cells and lead to dry eye postoperatively [21, 22]. We did not use any topical eye drops containing preservatives postoperatively for our patients not to increase dry eye symptoms.

We did not divide the patients into subgroups according to their degree of dry eye severity. We evaulated the patients as one group statistically. The use of means of this group’s test results might mask subgroups which might behave differently from the group as a whole. That was our limitation in this study.

Khanal et al. [9] reported that deterioration in corneal sensitivity and tear physiology was seen immediately after phacoemulsification. Corneal sensitivity didn’t return to preoperative levels until 3 months postoperatively whereas the tear functions recovered within 1 month. Kasetsuwan et al. [20] reported that, signs and syptoms of dry eye occured as early as 7 days post-phacoemulsification and the severity pattern improved over time. In our study also, dry eye test values returned to preoperative values after postoperative 3rd month.

Oh et al. [19] reported that, the decrease in goblet cell density, which was correlated with operation time, had not recovered at 3 months after cataract surgery, therefore, microscopic ocular surface damage during cataract surgery seems to be one of the pathogenic factors that causes ocular discomfort and dry eye syndrome after cataract surgery. Han et al. [23] reported that Meibomian gland function may be altered without accompanying structural changes after cataract surgery.

Movahedan et al. [24] reported that, maintaining a healthy ocular surface is essential for achieving the best visual outcome in cataract patients. Ocular surface preparation is beneficial not only in patients with established ocular surface disease, but also in those with minimal signs or symptoms of surface disease. Chung et al. [25] suggested that, cyclosporine 0.05 % can be an effective treatment for dry eye after cataract surgery.

## Conclusion

Phacoemulsification surgery may aggravate the signs and symptoms of dry eye and affect dry eye test values in chronic dry eye patients in short-term. However, in long-term, signs and symptoms of dry eye decrease and dry eye test values return to preoperative values.

## Additional file

Additional file 1:
**STROBE Statement.**

## Abbrevations

SD: Standard Deviation
OSDI: Ocular Surface Disease Index
BUT: Break-up Time
ST1: Schirmer Test 1
