# Supplementary material for: The course of dry eye after phacoemulsification surgery
Source: BMC Ophthalmol. 2015 Jun 30;15:68. doi: 10.1186/s12886-015-0058-3 (PMC4485332; doi:10.1186/s12886-015-0058-3)
Supplement: Additional file 1: — STROBE Statement. [file 12886_2015_58_MOESM1_ESM.docx]

STROBE Statement:

|  | Item No |  |
| --- | --- | --- |
| **Title and abstract** | 1 | **The Course of Dry Eye After Phacoemulsification Surgery** |
|  |  | To evaluate the course of dry eye syndrome after phacoemulsification surgery. |
| Introduction | | |
| Background/rationale | 2 | Dry eye syndrome is a multifactorial disease characterized by dryness of the ocular surface due to tear deficiency and overevaporation. Some surgical interventions related to anterior segment may also cause dry eye and aggravate the symptoms in pre-existing dry eye, like PRK, LASIK and cataract surgery. |
| Objectives | 3 | In this study, we evaluated the course of dry eye syndrome after phacoemulsification surgery. |
| Methods | | |
| Study design | 4 | Retrospective study |
| Setting | 5 | One hundred and ninety-two eyes of 96 patients with chronic dry eye syndrome and cataract were enrolled in this study. They had undergone uneventful phacoemulsification and IOL implantation operation between January 2010 and March 2011. |
| Participants | 6 | Their mean age was 68.46±8.14 (SD) (56-83) years . Thirty of them (31%) were male and 66 (69%) were female. They all had bilateral cataracts. |
|  |  | The mean follow-up time was two years. |
| Variables | 7 | OSDI Scores,Fluorescein Staining,BUT,ST1 |
| Data sources/ measurement | 8,9 | Their full ophthalmological examinations were performed 1 week before the surgery and 1^st^ day, 1^st^ week, 1^st^ month, 3^rd^ month, 6^th^ month, 1^st^ year and 2^nd^ year after the surgery and additionally fluorescein staining, BUT and ST1 without anesthesia were performed owing to the chronic dry eye. OSDI questionnaire was applied 1 week before the surgery and 1^st^ week,1^st^ month,3^rd^ month and 6^th^ month after the surgery.Fluorescein staining was classified according to Oxford Schema (Grade 0 to 5) |
|  |  |  |
| Study size | 10 | One hundred and ninety-two eyes of 96 patients with chronic dry eye syndrome and cataract were enrolled in this study. The mean follow-up time was two years. |
| Quantitative variables | 11 | OSDI Scores,Fluorescein Staining,BUT,ST1 |
| Statistical methods | 12 | SPSS version 22 programme was used. |
|  |  | For comparison of the data, Chi-square test and Paired t test were used. |
|  |  | A *P*<0.05 value was accepted as statistically significant. |
|  |  |  |
|  |  |  |
| Results | | |
| Participants | 13 | One hundred and ninety-two eyes of 96 patients with chronic dry eye syndrome and cataract were enrolled in this study. They had undergone uneventful phacoemulsification and IOL implantation operation. |
|  |  |  |
|  |  |  |
| Descriptive data | 14 | Their mean age was 68.46±8.14 (SD) (56-83) years . Thirty of them (31%) were male and 66 (69%) were female. |
|  |  | The mean follow-up time was two years. |
|  |  |  |
| Outcome data | 15 | OSDI scores were under 25 in 87 (91%) patients and between 25 and 30 in 9 (9%) patients preoperatively. However, postoperatively in 1^st^ week, it was under 25 in 15 (16%) patients, between 25 and 30 in 33 (34%) patients, between 30 and 40 in 21 (22%) patients and between 40 and 50 in 27 (28%) patients. In postoperative 1^st^ month, it was under 25 in 30 (31%) patients, between 25 and 30 in 39 (41%) patients and between 30 and 40 in 27 (28%) patients. In postoperative 3^rd^ month it was under 25 in 84 (88%) patients and between 25 and 30 in 12 (12%) patients. In postoperative 6^th^ month it was under 25 in 90 (94%) patients and between 25 and 30 in 6 (6%) patients. |
| Main results | 16 | According to Oxford Schema, preoperatively only 15 eyes had grade 2 fluorescein staining (7%). But postoperatively on 1^st^ day, 36 eyes had grade 2 (18%), 24 eyes had grade 3 (12%) and 12 eyes had grade 4 (6%) fluorescein staining pattern. In 1^st^ week 24 eyes had grade 2 (12%) and 12 eyes had grade 3 (6%) staining. In 1^st^ month 18 eyes had grade 2 (9%) and 6 eyes had grade 3 (3%) staining. In 3^rd^ month, only 6 eyes had grade 2 staining pattern (3%). |
|  |  | The mean preoperative BUT value was 11.65±2.31 (SD) (7-16) seconds. Postoperative 1^st^ day value was 7.60±1.24 (SD) (5-11), 1^st^ week value 7.03±0.97 (SD) (5-9), 1^st^ month value 7.42±0.79 (SD) (6-8), 3^rd^ month value 11.76±2.08 (SD) (9-16), 6^th^ month value 12.01±2.05 (SD) (9-16), 1^st^ year value 11.85±2,01 (SD) (8-17) and 2^nd^ year value was 11.95±1.92 (SD) (9-17) seconds. In comparison with preoperative value, the 1^st^ day, 1^st^ week and 1^st^ month values were significantly lower (*P*<0.001, *P*<0.001,*P*<0.001), however 3^rd^ month, 6^th^ month, 1^st^ year and 2^nd^ year values were not significantly different from preoperative value (*P*=0.441, *P*=0.078, *P*=0.145, *P*=0.125). |
|  |  | The mean preoperative ST1 value was 6.39±1.42 (SD) (4-9) mm. Postoperative 1^st^ day value was 4.59±1.06 (SD) (3-7), 1^st^ week value 4.45±0.95 (SD) (2-6), 1^st^ month value 4.50±1.00 (SD) (3-6), 3^rd^ month value 6.42±1.31 (SD) (4-9), 6^th^ month value 6.46±1.28 (SD) (4-10), 1^st^ year value 6.59±1.38 (SD) (4-9) and 2^nd^ year value was 6.54±1.29 (SD) (4-9) mm. In comparison with preoperative value, 1^st^ day, 1^st^ week and 1^st^ month values were significantly lower (P<0.001, *P*<0.001, *P*<0.001 ), however 3^rd^ month, 6^th^ month, 1^st^ year and 2^nd^ year values were not significantly different from preoperative value (*P*=0.748, *P*=0.439, *P*=0.091, *P*=0.214 ). |
|  |  |  |
| Discussion 17 | | |
| Key results | 18 | The subjective symptoms of the patients related to dry eye increased postoperatively. But after postoperative 1^st^ month, their complaints decreased gradually |
| Limitations | 19 | We did not divide the patients into subgroups according to their degree of dry eye severity. We evaulated the patients as one group statistically. The use of means of this group’s test results might mask subgroups which might behave differently from the group as a whole. That was our limitation in this study. |
| Interpretation | 20 | Phacoemulsification surgery may aggravate the signs and symptoms of dry eye and affect dry eye test values in chronic dry eye patients in short-term. However, in long-term, signs and symptoms of dry eye decrease and dry eye test values return to preoperative values. |
|  |  |  |
| Other information | | |
| Funding | 21 | The authors report no conflicts of interest. The authors alone are responsible for the content and writing of the paper. |
